# Metabolomics analysis of five cultivars of *Sorghum bicolor* reveals the contribution of flavonoid pathway to tannin content

**DOI:** 10.1371/journal.pone.0321649

**Published:** 2025-04-09

**Authors:** Xianlin Ni, Wenjing Long, Lijuan Gong, Jia Zheng, Yanghua Li, Zhenhui Kang

**Affiliations:** 1 Rice and Sorghum Research Institute, Sichuan Academy of Agricultural Sciences, Deyang, China; 2 Sichuan Sub Center, National Sorghum Improvement Center, Luzhou, China; 3 College of Biological Engineering, Sichuan University of Science & Engineering, Yibin, China; 4 Wuliangye Yibin Co., Ltd., Yibin, China; University of Brescia: Universita degli Studi di Brescia, ITALY

## Abstract

This study aims to utilize metabolomics to elucidate the key metabolites and metabolic pathways contributing to the quality differences among the grains of ‘hongyingzi’ (hyz) sorghum and four other varieties naming ‘jinuoliang’ (Jinl), ‘jinnuoliang’ (Jinnl), ‘lunuohong’ (lnh) and ‘liaoza 19’ (lz19). By analyzing the metabolomics data of the five sorghum varieties, we identified a total of 384 differentially accumulated metabolites (DAMs). Among these, flavonoids, phenolic acids and lipid compounds were the most abundant, exhibiting varying degrees of upregulation and downregulation. Compared to the four cultivars, the hyz sorghum differently exhibited 78, 40, 59 and 63 flavonoids; 29, 54, 30 and 30 phenolic acids; and 9, 27, 26 and 20 lipids, respectively. Multiple comparisons among the five sorghum varieties also identified 38 common DAMs, and the flavonoid pathway is particularly significant in KEGG enrichment. Additionally, as a high tannin content variety, correlation analysis revealed in sorghum that ellagic acid-4-O-glucoside plays a central role in tannin synthesis. These findings would highlight significant differences in the metabolomic profiles between hyz and the control varieties, providing valuable insights for the exploration of key genes involved in flavonoid metabolism and biosynthesis pathways in sorghum seeds.

## Introduction

Sorghum (Moench) is the fifth largest cereal crop globally, extensively cultivated in tropical, subtropical, and semi-arid regions. Compared to other grains, sorghum is rich in phenolic substances and tannins [1]. Tannin, a natural polyphenolic compound, is widely present in grains, berries, and tea, known for its strong antioxidant properties and ability to scavenge free radicals [2]. Tannins are categorized into hydrolyzable tannins (HT) and condensed tannins (CT), with sorghum containing CT [3]. Condensed tannins, also referred to as proanthocyanidins (PAs), serve as one of the colorants in plants [4]. Structurally, condensed tannins belong to the C6-C3-C6 structural type polyphenols, and polymers are composed of Flavan-3-ols as structural units [5]. Proanthocyanidins impart a brown color to the seed coat of sorghum and other seeds. During the brewing process, proanthocyanidins significantly influence the taste and flavor quality of Chinese *baijiu* [6].

Tannins, a type of flavonoid secondary metabolite, are synthesized under the regulation of structural genes and transcriptional factors [7]. These structural genes include early and late structural ones [8], while the bHLH and WD40 transcriptional factors are the primary regulators of the anthocyanin synthesis pathway [9]. These proteins typically regulate the synthesis of anthocyanins and proanthocyanidins through the formation of ternary complexes known as MYB-bHLH-WD40 (MBW) complexes. The biosynthesis of anthocyanins involves four main biochemical pathways, which are the shikimic acid pathway, the phenylpropane pathway, the flavonoid pathway, and the anthocyanin synthesis pathway[10]. The presence of tannins in sorghum grains is influenced by different alleles of the *Tannin1* gene [11]. Recently, three new recessive alleles regulating sorghum tannin synthesis were identified at the *TAN1* and *TAN2* locus [12].

Tannins in sorghum serve as crucial indicators for assessing the quality of grains. Despite the potential to impair the digestibility of starch and protein, tannin plays a beneficial role in Chinese *baijiu* brewing. Specifically, tannin concentrations ranging from 0.5% to 1.5% can inhibit the growth of mixed bacteria during brewing and generate unique aromatic compounds, such as syringic acid and syringaldehyde during fermentation. These compounds directly influence the taste and quality of the Chinese *baijiu* [13,14]. Tannins are predominantly located in the seed coat of sorghum, with their content varying across different varieties. Pigment deposition results in diverse grain colors within the pericarp, seed coat, and endosperm of sorghum grains [15]. Sorghum varieties are categorized based on tannin presence and accumulation location. The type I sorghum lacks tannins or a seed coat layer; type II sorghum contains tannins that accumulate in vesicles within the seed coat layer; and type III sorghum tannins deposit in the cell wall of the seed coat, forming what is known as the tannin layer [16]. The color of the sorghum seed coat is typically positively correlated with pigment content; higher pigment levels result in darker grain colors. Pigments, inherited as quantitative traits, are controlled by multiple major coloring seed coat genes. Additionally, the color and thickness of the fruit coat, along with the presence or absence of the seed coat, influence seed color. Tannin presence in sorghum is determined by two dominant complementary genes, B1 and B2. A homozygous recessive mutation in either gene can result in tannin deficiency [17].

Due to the ability of sorghum phenolic extracts to prevent chronic human diseases such as obesity, cancer, and Alzheimer’s Disease [18,19], studies have utilized techniques such as Genome-Wide Association Studies (GWAS) to explore quantitative trait locus and key genes involved in sorghum polyphenol synthesis [20]. Sorghum can activate or modify certain small molecule metabolites in response to biotic or abiotic stress, and metabolomics and other technical methods can provide important information on related metabolic pathways [21,22]. Widely targeted and non-targeted metabolomics can determine the effects of genetic, physiological, and ecological factors on various secondary metabolites in crops [8,23–27]. These studies have found that sorghum is rich in various polyphenolic compounds, such as anthocyanins and 3-deoxyanthocyanins, which impart excellent antioxidant activity to sorghum-related functional foods and animal feed. However, the brewing characteristics of sorghum necessitate large-scale detection, identification, and quantification of all chemical and nutritional components in sorghum grains to provide a theoretical basis for the metabolic characteristics of different brewing sorghum varieties. For example, ‘hongyingzi’ (hyz), a sorghum variety used for brewing Maotai liquor, contains 83.40% of total starch, a 96.27% of amylopectin/total starch ratio, and 1.61% tannins. Whole genome resequencing of hyz had revealed 35 gene variations related to tannin synthesis [28]. Proteomics and metabolomics studies indicate that abundant α-glucosidase enhances the fermentability of sorghum in beer brewing [29]. In this study, a widely non-targeted metabolomics method based on UPLC-ESI-MS/MS was employed to identify and quantify the primary and secondary metabolites of different colored sorghum grains. The hyz was selected as the representative variety with high tannin content, while four other representative sorghum varieties with different pigments and moderate quality were chosen as controls. Metabolomics was used to study the differential metabolites of these five sorghum varieties, aiming to investigate the metabolic changes responsible for pigment variation in sorghum seeds. The research results provide a theoretical basis for understanding the synthesis mechanisms of sorghum.

## 2. Materials and methods

### 2.1 Materials and cultivation

This study selected five representative sorghum varieties with different seed colors, namely ‘hongyingzi, hyz’ and ‘Jinnuoliang, jinnl’, ‘Liaoza19, lz19’, ‘Lunuohong, lnh’, ‘Jinuoliang, jinl’. All sorghum seeds were sown in Luzhou, Sichuan, China (N27°39’-29°20’, E105°08’-106°28’) using a randomized complete block design, with 16 rows planted in each plot under the same cultivation environment. The field experiments were conducted on the farm of the field practice base with permission of the Rice and Sorghum Research Institute of the Sichuan Academy of Agricultural Sciences (Luzhou). After five months of growth, the experiment was repeated three times. During the flowering period, the sorghum was bagged, and the bags were replaced with net bags at the end of the flowering period. After reaching maturity, the sorghum was harvested. All sorghum varieties were sown on April 2 and 6, 2023, and mature grains without damage or mold were randomly harvested from the plants. After vacuum freeze-drying for 24 h, all materials were immediately frozen in liquid nitrogen and stored at -80°C until further use for metabolomics research. Three individual plants were used for each sample, and different qualities of sorghum grains were mixed to produce quality control (QC) samples.

### 2.2 Sample preparation and extraction

Biological samples were freeze-dried using a vacuum freeze-dryer (Scientz-100F). The freeze-dried samples were then crushed using a mixer mill (MM 400, Retsch) with a zirconia bead for 1.5 min at 30 Hz. A total of 100 mg of the lyophilized powder was dissolved in 1.2 mL of 70% methanol solution, vortexed for 30 s every 30 min for a total of six times, and then placed in a refrigerator at 4°C overnight. Following centrifugation at 12,000 *g* for 10 min, the extracts were filtered with SCAA-104 (0.22 μm) (ANPEL, Shanghai, China) before UPLC-MS/MS analysis.

### 2.3 UPLC conditions

The sample extracts were analyzed using an UPLC-ESI-MS/MS system (UPLC, Shimadzu, Nexera X2; MS, Applied Biosystems, 4500 Q TRAP). An Agilent SB-C18 (1.8 µm, 2.1 mm*100 mm) was used as the UPLC column. The mobile phase was consisted of solvent A (formic acid, 0.1%), and solvent B, that is acetonitrile with 0.1% formic acid. Sample measurements were performed with a gradient program that employed the starting conditions as a ratio of 95% to 5% for the solvent A and solvent B. Within 9 min, a linear gradient to 5% solvent A and 95% solvent B was programmed, and a composition of 5% solvent A and 95% solvent B was kept for 1 min. From the tenth to eleventh min, the proportion of phase B decreases to 5% and equilibrates at 5% until the fourteenth min. The flow rate is 0.35 mL min^-1^; the column temperature is 40°C, and the injection volume is 4 μl.

### 2.4 ESI-Q TRAP-MS/MS

Mass spectrometry detection was mainly operated on a AB4500 Q TRAP UPLC/MS/MS System being controlled by Analyst 1.6.3 software. The experimental procedure was carried out according to Zhou et al., (2022) with minor modification.

### 2.5 Principal component analysis (PCA)

Unsupervised PCA was performed by statistics function prcomp within R package (www.r-project.org). The data was unit variance scaled before unsupervised PCA.

### 2.6 Hierarchical Cluster Analysis (HCA) and Pearson Correlation Coefficients (PCC)

The HCA results of samples and metabolites were presented as heatmaps with dendrograms, while PCC among samples was calculated by the core function in R and presented as only heatmaps. Both HCA and PCC were carried out by R package heatmap. For HCA, normalized signal intensities of metabolites (unit variance scaling) are visualized as a color spectrum.

### 2.7 Differential metabolites selected

Significantly regulated metabolites between groups were determined by VIP>= 1 and absolute Log_2_FC (fold change)>=1. VIP values were extracted from OPLS-DA results, which also contain score plots and permutation plots, and were generated using the R package MetaboAnalystR. The data was log transform (log2) and mean centering before OPLS-DA. To avoid overfitting, a permutation test (200 permutations) was performed.

### 2.8 KEGG annotation and enrichment analysis

Identified metabolites were annotated using the KEGG Compound database (http://www.kegg.jp/kegg/compound/), and annotated metabolites were then mapped to the KEGG Pathway database (http://www.kegg.jp/kegg/pathway.html). Pathways with significantly regulated metabolites mapped to were then fed into metabolite sets enrichment analysis (MSEA), their significance was determined by the hypergeometric test’s *p*-values.

### 2.9 Content measurement of total phenolic acids, flavonoids and tannins

The content of total phenolic acids, flavonoids was measured according to [30], and the tannin content was determined with the methods provided by [16].

### 2.10 Statistical analysis

Data processing was conducted using SPSS software and significance analysis was performed using student’s *t*-test (*P*<0.05).

## 3. Results

3.1 The contents of total phenol, flavonoid, and tannin were significantly different among the five sorghum varieties

Firstly, the content of total phenol, flavonoid, and tannin should be measured ahead to ensure the reliability of subsequent metabolic analysis. It is crucial to acknowledge that there were significant differences in the contents of total phenolic acids, total flavonoids, and tannins among the five sorghum varieties (Table 1). The total flavonoids in hyz, lnh, jinnl, jinl and lz19 were 555.36±30.57, 592.24±16.93, 423.57±25.66, 449.62±23.81 and 271.48±18.04 mg 100 g^-1^ respectively. The total phenolic acids were 1671.58± 20.51, 1546.84± 15.34, 1232.06±15.86, 1035.39±18.42, and 908.93±10.67 mg 100 g^-1^ in the five varieties. Total tannins in the grains of the five sorghum was 1.75±0.14%, 1.70±0.12%, 1.36±0.10%, 1.30±0.08%, and 1.12±0.09% as calculated to dry weight. This recognition will help in designing experiments with appropriate controls and replicates and accurately interpreting the results.

**Table 1:** Total content of phenolic acids, flavonoids and tannins in five sorghum cultivars.

variety	Total flavonoids (mg 100 g^-1^)	Total phenolic acids (mg 100 g^-1^)	Total tannins
hyz	555.36±30.57 (a)	1671.58± 20.51 (a)	1.75±0.14% (a)
lnh	592.24±16.93 (b)	1546.84± 15.34 (b)	1.70±0.12% (a)
jinnl	423.57±25.66 (c)	1232.06±15.86 (c)	1.36±0.10% (b)
jinl	449.62±23.81 (d)	1035.39±18.42 (d)	1.30±0.08% (b)
lz19	271.48±18.04 (e)	908.93±10.67 (e)	1.12±0.09% (c)

All data was listed as Mean±SD (standard deviation). The content was determined in seeds from six independent sorghum plants of each variety (n=6). The different letters in each column indicate significant differences (*P*<0.05) within the group.

### 3.2 Evaluation of quantitative analysis of metabolites

**Fig 1:**
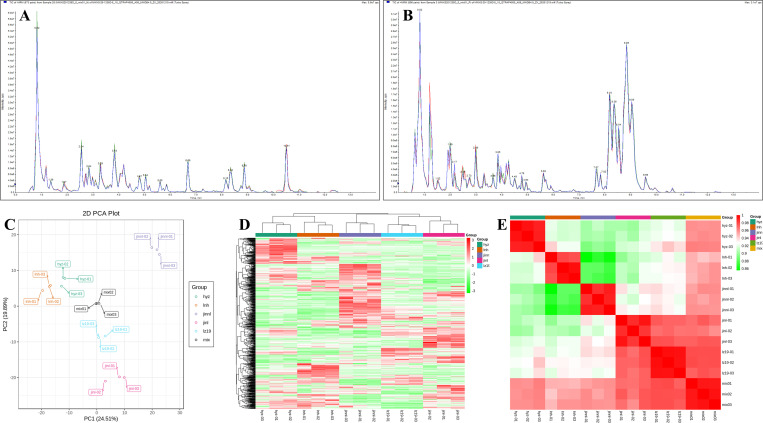
Evaluation of metabolomics data. **Sample quality control analysis was used to analyze the repeatability of samples under the same condition in negative ion mode.** (A) and positive ion mode (B). The total ion current (TIC) of the mixed quality control (QC) sample is a continuous plot of the intensity of all ions in the mass spectrum at each time point, with the horizontal axis representing the retention time (Rt) for metabolite detection and the vertical axis representing the ion current intensity for ion detection. (C) PCA score plot of mass spectrometry data for each group of samples and QC samples. PC1 and PC2 represents the first and second principal component, and percentages represent the explanatory power of these principal components on the dataset; Each point in the figure represents a sample, and samples from the same group are represented by the same color. (D) Cluster heatmap analysis on all samples with R program. Different colors represent values obtained after relative content standardization. (E) The R software is used to calculate the value of r, of which r2 is closer to 1, the correlation is stronger.

The composition of related metabolites and differentially accumulated metabolites (DAM) in the grains of five sorghum varieties was determined using the UPLC-ESI-MS/MS widely non-targeted metabolomics method. The repeatability of metabolite extraction and detection was assessed using the total ion chromatogram (TIC) of mass spectrometry detection and analysis of various quality control (QC) samples. The QC demonstrated a high degree of curve overlap in metabolite detection, indicating consistent retention times and peak intensities. This consistency signified that mass spectrometry provides stable signal detection for the same sample across different times (Fig 1A and 1B).

Principal component analysis (PCA) of the samples, including QC samples, revealed the separation trends within the metabolome groups. The PCA results indicated that the metabolomic differences between samples were relatively minor (Fig 1C). Hierarchical cluster analysis (HCA) was performed on normalized data to generate cluster heatmaps using R scripts. As illustrated in Fig 1D, individuals within the same category displayed high homogeneity, while distinct categories exhibited significant heterogeneity.

Furthermore, correlation analysis between samples showed strong biological replicates within groups. The Pearson correlation coefficient ranged from 0.86 to 1, underscoring the reliability of differential metabolites identified between repeated samples within the same group (Fig 1E). These findings confirmed that the metabolites identified through mass spectrometry screening are suitable for subsequent differential analysis.

### 3.3 PCA and OPLS-DA analysis of hyz and control sorghum varieties

**Fig 2:**
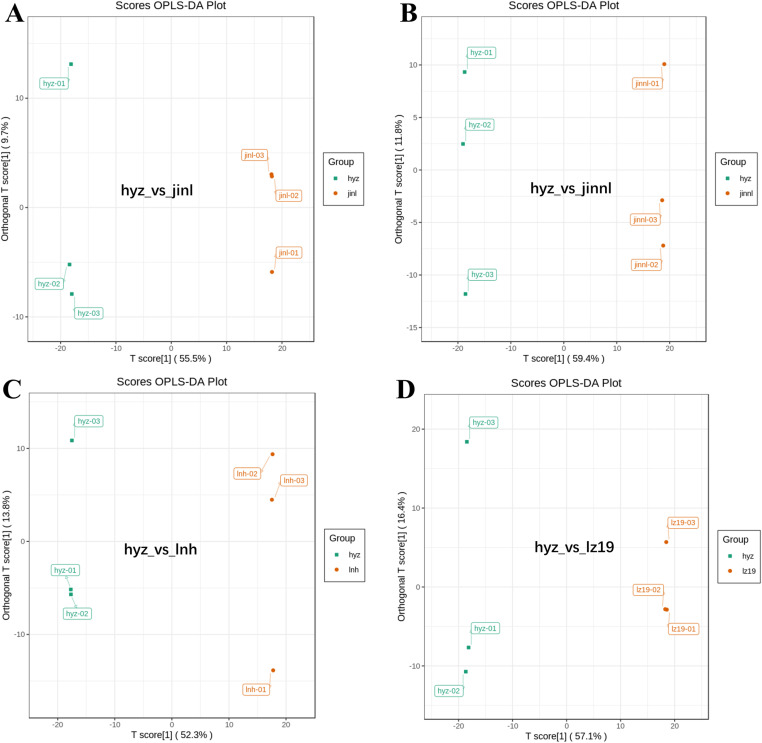
The PCA analysis of the four groups of sorghum varieties respectively. (A) hyz_vs_jinl. (B) hyz_vs_jinnl. (C) hyz_vs_lnh. (D) hyz_vs_lz19. Each group has a PCA graph, where PC1 represents the first principal component, PC2 represents the second principal component, and percentages represent the explanatory power of the principal component on the dataset; Each point in the figure represents a sample, and samples in the same group are represented by the same color. Group represents grouping.

Prior to analyzing differential metabolites, the PCA analysis was performed on the grouped samples being subjected to differential comparison analysis to observe the degree of variation between and within groups. The cumulative contribution rates of the two principal components (PC1: 55.34% and PC2: 18.52%; PC1: 59.18 and PC2: 15.04%; PC1: 52.52% and PC2: 18.2%; PC1: 57.07% and PC2: 16.76%) in the PCA score chart reached to 73.86%, 74.22%, 70.72% and 73.83% for groups of hyz_vs_jinl, hyz_vs_jinnl, hyz_vs_lnh and hyz_vs_lz19 respectively. As illustrated in Fig 2, the five sorghum varieties were well separated each pair, indicating significant differences in their metabolic profiles. Furthermore, each of the three biological replicates for each variety formed a compact cluster, demonstrating significant intragroup consistency. These results suggest pronounced metabolic differences among the different sorghum varieties, making them suitable for subsequent qualitative and quantitative analysis.

An orthogonal partial least squares discriminant analysis (OPLS-DA) model was used to compare differentially accumulated metabolites (DAMs) among the sorghum varieties. This study employed a pairwise comparison method based on the OPLS-DA model to evaluate all metabolites in sorghum grains. The OPLS-DA model was used to analyze the metabolomic data, generate score plots for each group, and further illustrate the differences between the groups. The predictive parameters of the evaluation model include R2X, R2Y, and Q2, where R2X and R2Y represent the explanatory power of the model for the X and Y matrices, respectively, and Q2 represents the predictive ability of the model. The closer these three indicators are to 1, the more stable and reliable the model is. A Q2 value greater than 0.5 indicates an effective model, while a Q2 value greater than 0.9 indicates an excellent model.

**Fig 3:**
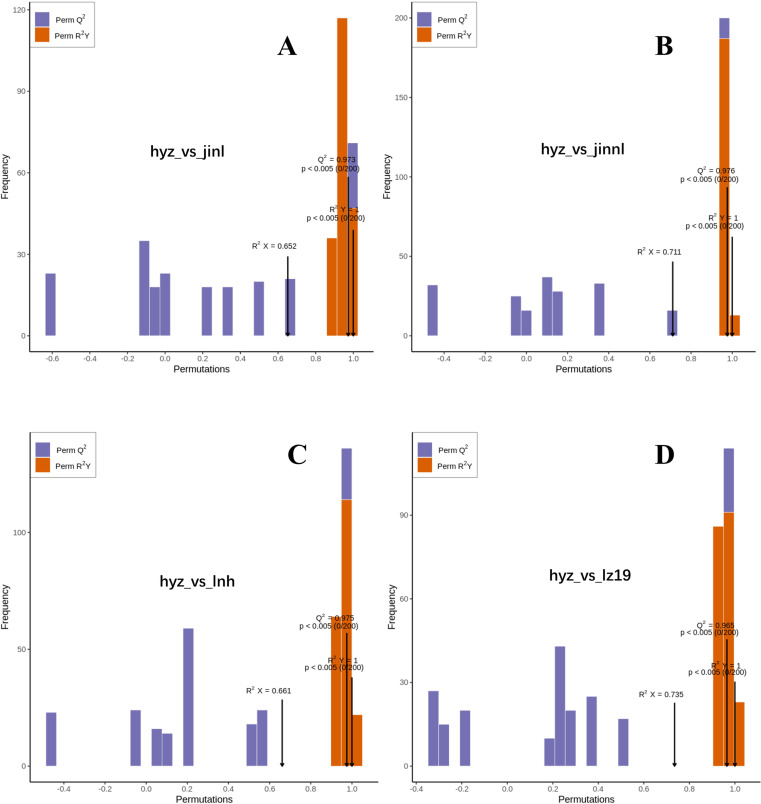
The OPLS-DA analysis of four groups of sorghum varieties respectively. (A) hyz_vs_jinl_opls_score. (B)hyz_vs_jinnl_opls_score. (C) hyz_vs_lnh_opls_score. (D) hyz_vs_lz19_opls_score. The horizontal axis represents the covariance between principal components and metabolites, while the vertical axis represents the correlation coefficient between principal components and metabolites. Metabolites closer to the upper right and lower left corners indicate more significant differences. Red dots indicate that the VIP value of these metabolites is greater than or equal to 1, while green dots indicate that the VIP value of these metabolites is less than 1.

This study utilized the OPLS-DA model to compare all metabolites of different sorghum grains in pairs, determining the differences between hyz_vs_jinl (Q2=0.973, Fig 3A), hyz_vs_jinnl (Q2=0.976, Fig 3B), hyz_vs_lnh (Q2=0.975, Fig 3C), and hyz_vs_lz19 (Q2=0.965, Fig 3D). The Q2 values of all four comparison groups were greater than 0.9, indicating that these models are highly steady to provide a robust explanation for the metabolic changes among the five varieties. Differentially accumulated metabolites can be further identified through subsequent variable importance in projection (VIP) analysis.

### 3.4 DAMs classification of hyz and control sorghum varieties

Based on the OPLS-DA results, the variable importance in projection (VIP) of the obtained multivariate analysis OPLS-DA model was used to preliminarily screen DAMs between different pairs. At the same time, the *p*-value or fold change of univariate analysis was combined to further screen for differential metabolites. In this study, three biological replicates were set for each sample, and a combination of fold change and VIP values from the OPLS-DA model was used to screen for differential metabolites. Metabolites with fold change ≥ 2 and fold change ≤ 0.5 were selected primarily. If the difference in metabolites between the control group and the experimental group is more than twice or less than 0.5, the difference is considered to be significant. Metabolites with VIP ≥ 1 were also selected, and the VIP value represents the strength of the impact of the inter group differences of the corresponding metabolites on the classification of each group of samples in the model. It is generally believed that metabolites with VIP ≥ 1 have significant differences.

In this study, a total of 713 metabolites were identified from 11 major categories of 5 sorghum varieties, including 376 primary metabolites (lipids, amino acids and derivatives, nucleotides and derivatives, organic acids, and others) and 337 secondary metabolites (flavonoids), phenolic acids, alkaloids, terpenoids, lignans and coumarins, tannins) (Table S1). However, no steroid substances were detected in the secondary metabolites. The classification and quantity of these metabolites were flavonoids (142), lipids (132), phenolic acids (117), other classes (72), organic acids (64), amino acids and their derivatives (61), alkaloids (56), nucleotides and their derivatives (47), lignin and coumarins (10), tannins (10), and terpenes (2) in order, respectively. It can be seen that phenolic acids, flavonoids, and lipids account for the highest proportion. All identified metabolites can be found in Table S1. According to the HCA heatmap, the 15 samples can be clearly divided into 5 groups, indicating strong differences in metabolite profiles among different sorghum varieties.

We found that among these 11 types of compounds, each major group of compounds has different glycosylation modifications, covering almost all possible glycosylation sites. Among the 142 identified flavonoids, there are a total of 6 chalcones, 12 dihydroflavones, 5 dihydroflavones, 7 flavanols, 59 flavonoids, 13 flavonoid carbonyl, 30 flavonols, 9 isoflavones, and 1 sinensetin. Among these, 11 types of flavonoids are modified by glucose and arabinose glycosidic bonds, and are mainly glycosylated at positions 8-C, 6-C, and 7-O. Fifteen phenolic acids are modified with glycosidic bonds, mainly glucosides, which are characterized by only containing O-glycosidic bonds but not C-glycosidic bonds.

### 3.5 Key DAMs in grains of hyz and control sorghum varieties

**Fig 4:**
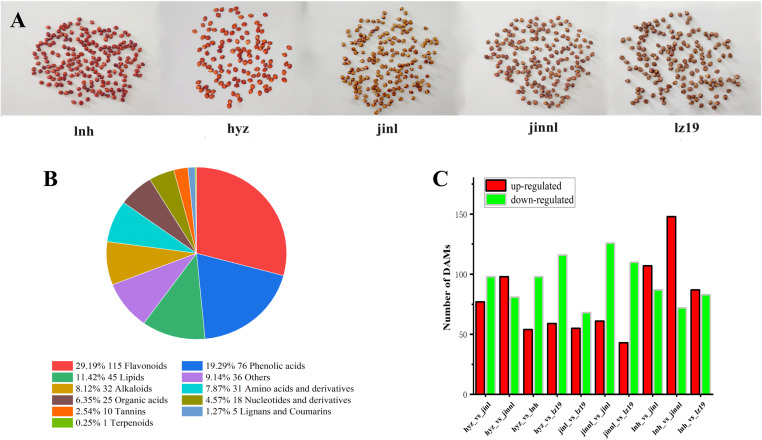
Comparison of total DAMs and differences among five sorghum varieties. (A) Photograph of five sorghum varieties. (B) Total number and classification ratio of DAMs. (C) Analysis of differences in DAMs among different varieties.

The five sorghum varieties selected for this study are hyz, jinnl, lnh, jinl, and lz19 (Fig 4A). The differential metabolites (DAMs) of these sorghum varieties were analyzed through four pairwise comparison groups, encompassing 11 classifications: alkaloids, amino acids and derivatives, flavonoids, lignans and coumarins, lipids, nucleotides and derivatives, organic acids, phenolic acids, tannins, terpenoids, and others (Fig 4B). The comparison between hyz and jinl revealed 175 DAMs (98 upregulated and 77 downregulated) across 10 categories. Similarly, the comparison between hyz and jinnl identified 179 DAMs (81 upregulated and 98 downregulated) across 11 categories; hyz and lnh exhibited 152 DAMs (98 upregulated and 54 downregulated) across 9 categories; hyz and lz19 had 175 DAMs (116 upregulated and 59 downregulated) across 11 categories; jinl and lz19 presented 123 DAMs (68 upregulated and 55 downregulated); jinnl and lz19 displayed 153 DAMs (110 upregulated and 43 downregulated); lnh and jinl showed 194 DAMs (87 upregulated and 107 downregulated); lnh and jinnl had 220 DAMs (72 upregulated and 148 downregulated); and lnh and lz19 revealed 170 DAMs (83 upregulated and 87 downregulated) (Fig 4C). Among these DAMs, flavonoids, phenolic acids, and lipids were the most abundant categories. Notably, compared to the other varieties, hyz exhibited predominantly downregulated lipids, indicating a lower fatty acid content, consistent with its optimal brewing characteristics.

**Fig 5:**
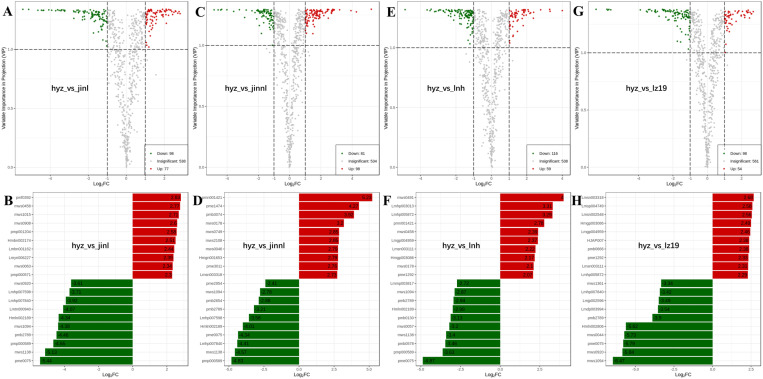
Metabolite differences between hyz and control varieties. The volcano plots in the A, C, E and G plots show different levels of metabolite expression between pairs of hyz_vs_jinl, hyz_vs_jinnl, hyz_vs_lnh and hyz_vs_lz19. Red dots, green dots, and gray dots represent upregulated, downregulated, and insignificant differentially expressed metabolites, respectively. the B, D, F and H are the top 10 compounds upregulated and downregulated among the samples of hyz_vs_jinl, hyz_vs_jinnl, hyz_vs_lnh and hyz_vs_lz19, respectively.

Qualitative and quantitative analysis of DAMs between hyz and control sorghum varieties revealed that the most significantly upregulated and downregulated compounds were primarily flavonoids, phenolic acids, and lipids. It had been totally identified 175 DAMs between hyz and jinl sorghum with 98 downregulated and 77 upregulated (Fig 5A). Among these DAMs, there were 78 flavonoids with 29 upregulated and 49 downregulated (Table S2). Among the ten most significantly upregulated compounds, six DAMs were flavonoids, including persicogenin (5,3’-dihydroxy-7,4’-dihydroxyflavonone), glycitein, apigenin-7,4’-dihydroxyflavone, galangin (3,5,7-trihydroxyflavone), genistein, and apigenin, all of which are upregulated by more than 4.9 times. Four of the ten most significantly downregulated compounds were flavonoids, namely tricetin (5,7,3’,4’,5’-pentahydroxyflavone), quercetin-3-O-(6’-malonyl) galactoside, 13S-hydroperoxy-6Z,9Z,11E-octadecatrienoic acid, and quercetin-7-O-(6’-malonyl) glucoside, and all of which are downregulated by more than 3.6 times (Fig 5B). Compared to jinnl, there were 98 upregulated and 81 downregulated DAMs (Table S3), in which seven of the ten most significantly upregulated substances in hyz were phenolic acids, and four of the ten most significantly downregulated substances were flavonoids (Fig 5C and D). A total of 59 upregulated and 116 downregulated DAMs were identified in hyz compared to lnh (Table S4), in which seven of the ten most significantly upregulated substances were phenolic acids, and four of the ten most significantly downregulated substances were flavonoids (Fig 5E and F). By contrast to lz19, we found 54 upregulated and 98 downregulated DAMs (Table S5), among which six of the ten most significantly upregulated substances in hyz were flavonoids, four DAMs were phenolic acids, and eight of the ten most significantly downregulated substances were flavonoids (Fig 5G and H). These results indicate that, compared to the other four control sorghum varieties, the main DAMs in hyz belong to flavonoids and phenolic acids.

### 3.6 KEGG analysis of differential metabolites between grains of hyz and control sorghum varieties

**Fig 6:**
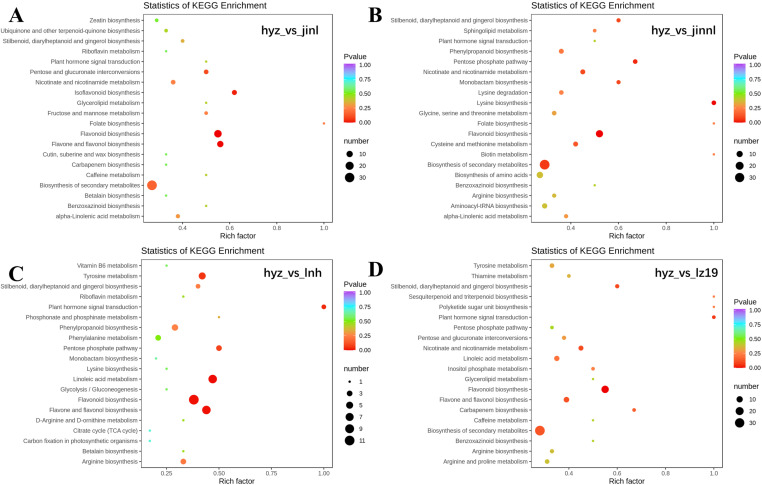
The KEGG pathway enrichment of differentially accumulated metabolites among different groups. (A) hyz_vs_jinl. (B) hyz_vs_jinnl. (C) hyz_vs_lnh. (D) hyz_vs_lz19. The horizontal axis represents the rich factor corresponding to each pathway, the vertical axis represents the pathway name, and the color of the points is *P*-value. The redder the points, the more significant the enrichment. The size of the dots represents the number of enriched differential metabolites.

Enrichment analysis of DAMs from the five samples using the KEGG database revealed key metabolic pathways related to tannins. The DAMs in the four comparison groups, hyz_vs_jinl, hyz_vs_jinnl, hyz_vs_lnh, and hyz_vs_lz19 were enriched in 56, 58, 42, and 62 pathways, respectively (Fig 6A-D). The proportion of DAMs involved in metabolic pathways was 60.27%, 69.86%, 61.4%, and 68.92%, and the proportion of compounds in secondary metabolic pathways was 43.84%, 46.58%, 35.09%, and 44.59%, respectively. A total of 20 metabolic pathways were identified, with 73, 73, 57, and 74 secondary metabolites exhibiting differences among the four comparison groups (Fig 6). In the hyz_vs_jinl group, flavonoid biosynthesis, flavone and flavonol biosynthesis, and isoflavonoid biosynthesis were the pathways with the highest number of DAMs, with 16, 10, and 5 DAMs, respectively. For the hyz_vs_jinnl group, the top pathways were flavonoid biosynthesis, ABC transporters, and aminoacyl tRNA biosynthesis, with 15, 7, and 6 DAMs, respectively. In the hyz_vs_lnh group, linoleic acid metabolism, flavonoid biosynthesis, and flavone and flavonol biosynthesis were the main pathways, with 8, 11, and 8 DAMs, respectively. For the hyz_vs_lz19 group, flavonoid biosynthesis, ABC transporters, and flavone and flavonol biosynthesis were the leading pathways, with 16, 7, and 7 DAMs, respectively.

In the hyz_vs_jinl group, five DAMs were upregulated in the flavonoid biosynthesis pathway: apigenin, galangin (3,5,7-trihydroxyflavone), myricetin, isosalipurposide (phloridzin chalcone), and naringenin-7-O-neohesperidoside (naringin). Additionally, afzelechin (3,5,7,4’-tetrahydroxyflavan) was downregulated. Eleven other flavonoids were downregulated, including dihydrokaempferol, eriodictyol (5,7,3’,4’-tetrahydroxyflavanone), epicatechin, catechin, tricetin (5,7,3’,4’,5’-pentahydroxyflavone), quercetin, dihydroquercetin (taxifolin), dihydromyricetin (ampelopsin), 5-O-p-coumaroylquinic acid, naringenin-7-O-glucoside (prunin). In the hyz_vs_jinnl group, six DAMs were upregulated, including luteoforol (3’,4’,5’,7-pentahydroxyflavan), 5-O-p-Coumaroylquinic acid, trans-5-O-(p-coumaroyl) shikimate, 3-O-caffeoylquinic acid (chlorogenic acid), isosalipurposide (phloridzin chalcone), and naringenin-7-O-neohesperidoside (naringin). Six DAMs were downregulated, including aromadendrin (dihydrokaempferol), eriodictyol (5,7,3’,4’-tetrahydroxyflavanone), epicatechin, catechin, tricetin (5,7,3’,4’,5’-pentahydroxyflavone), quercetin, dihydroquercetin (taxifolin), dihydromyricetin (ampelopsin), and apigenin-8-C-glucoside (vitexin). In the hyz_vs_lnh group, upregulated DAMs included chlorogenic acid (3-O-caffeoylquinic acid), isosalipurposide (phloridzin chalcone), and naringenin-7-O-neohesperidoside (naringin). Downregulated DAMs included aromadendrin (dihydrokaempferol), eriodictyol (5,7,3’,4’-tetrahydroxyflavanone), tricetin (5,7,3’,4’,5’-pentahydroxyflavone), quercetin, dihydroquercetin (taxifolin), dihydromyricetin (ampelopsin), 5-O-caffeoylshikimic acid, and naringenin-7-O-glucoside (prunin). For the hyz_vs_lz19 group, upregulated DAMs included 5-O-p-Coumaroylquinic acid, trans-5-O-(p-coumaroyl) shikimate, 3-O-caffeoylquinic acid (chlorogenic acid), and isosalipurposide (phloridzin chalcone). Downregulated DAMs included phloretin, afzelechin (3,5,7,4’-tetrahydroxyflavan), 3,4,2’,4’,6’-pentahydroxychalcone, aromadendrin (dihydrokaempferol), eriodictyol (5,7,3’,4’-tetrahydroxyflavanone), epicatechin, catechin, tricetin (5,7,3’,4’,5’-pentahydroxyflavone), quercetin, dihydroquercetin (taxifolin), dihydromyricetin (ampelopsin), and naringenin-7-O-glucoside (prunin).

### 3.7 Integrated analysis of DAMs in five sorghum varieties

**Fig 7:**
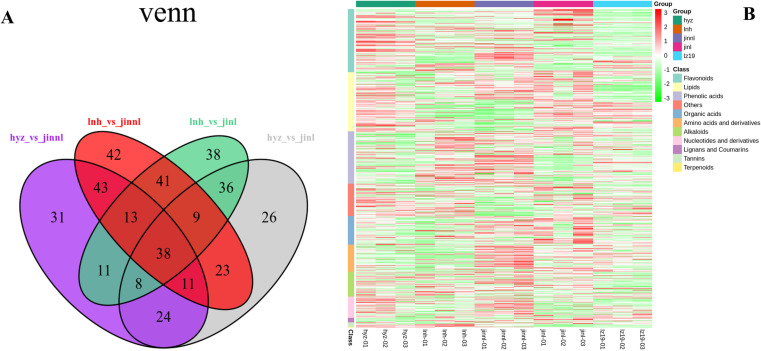
The five sorghum varieties integrated 384 DAMs in total. (A) The relationships among the differential metabolites of various groups through the form of a Venn diagram, in which the numbers represent the intersections of multiple comparisons. (B) The heatmap displays the clustering of 384 DAMs into a total of 11 major subclasses of secondary metabolites.

Conducting an integrated analysis of the total DAMs in five sorghum varieties will facilitate further identification of key metabolites. The relationships among the DAMs of each group were presented in the form of a Venn diagram. It had been shown that in the five sorghum varieties a total of 384 common and specific DAMs were revealed across all pairwise and multiple comparison groups (Fig 7A, Table S6). These 11 major categories of secondary metabolites, clustered from 384 DAMs by the heatmap, consisted of 32 alkaloids, 31 amino acids and derivatives, 87 flavonoids, 5 ligans and coumarins, 44 lipids, 16 nucleotides and derivatives, 25 organic acids, 73 phenolic acids, 10 tannins, 1 terpenoid, and 33 others separately (Fig 7B).

### 3.8 Representative DAMs were enriched in the flavonoid pathway

**Fig 8:**
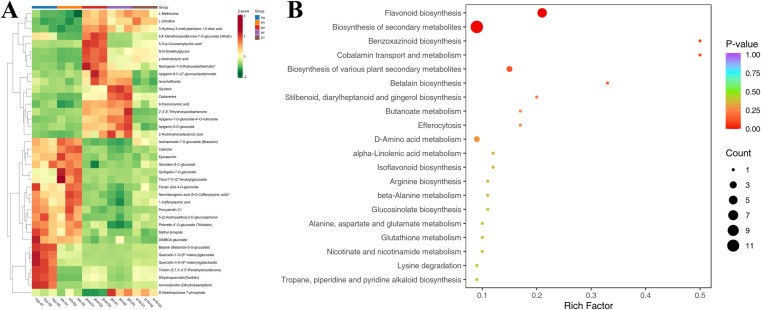
Venn analysis has identified 38 DAMs that are shared by the five sorghum varieties. (A) Heatmap of the 38 DAMs. (B) KEGG analysis revealed that the common DAMs are mainly enriched in the flavonoid pathway.

It is noteworthy that 38 DAMs common to all five sorghum varieties have been identified through Venn diagram analysis, leaving the remaining as specific DAMs to pairwise comparisons. The total count was 3 alkaloids, 4 amino acids and derivatives, 19 flavonoids, 3 organic acids, 1 other, 7 phenolic acids, and 1 tannin, indicating that flavonoids and phenolic compounds constitute the overwhelming majority (Table S7). These DAMs had been visualized through the generation of a heatmap (Fig 8A). If these common differential metabolites are analyzed within the context of hyz, we can identify 15 compounds that are upregulated, 17 compounds that are downregulated, and 7 compounds that show various levels. To investigate what pathways the shared DAMs interact within sorghums to form, annotation and visualization of these differential metabolites were conducted using the KEGG database. Statistic KEGG enrichment showed that the flavonoids occupied a prominent position in the top 20 biological pathways (Fig 8B).

We also conducted an analysis of compounds involved in tannin synthesis among all DAMs, especially in hyz. A total of 10 DAMs were identified, including 3-O-methylellagic acid, digallic acid, ellagic acid-4-O-glucoside, procyanidin A2, procyanidin A1, procyanidin B2, procyanidin B3, procyanidin B1, procyanidin C2, and procyanidin C1. Except for ellagic acid-4-O-glucoside whose content was upregulated, all other compounds showed varying degrees of reduction compared to the control group, suggesting that the upregulation of ellagic acid-4-O-glucoside is the key factor contributing to the high tannin content in hyz sorghum.

**Fig 9:**
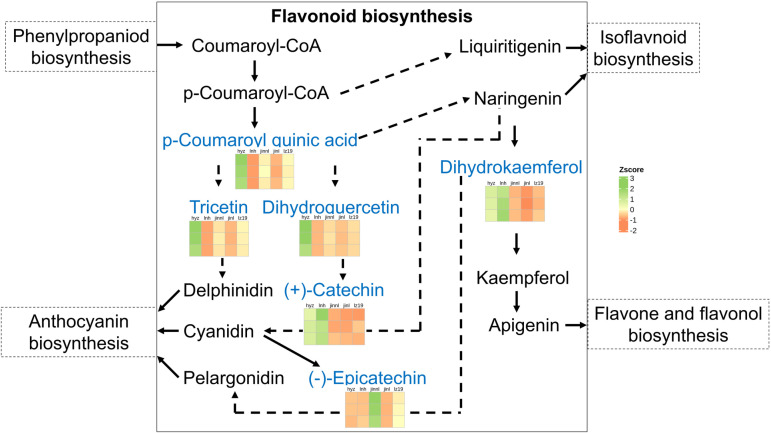
The flavonoid pathway holds greater significance in tannin metabolism, as it is the only pathway where differential metabolites were enriched. A heatmap visualization was subsequently conducted to represent these metabolites.

A total of six common DAMs were identified in the flavonoid pathway, and they were p-Coumaroyl quinic acid, Tricetin, Dihydrokaemferol, Dihydroquercetin, (+)-Catechin, and (-)-Epicatechin. Although anthocyanins can be synthesized through four pathways, the differential metabolites are only mapped to one of these pathways, namely the flavonoid pathway. Therefore, we created a schematic diagram that illustrated the six differential metabolites within the flavonoid pathway along with their corresponding heatmap (Fig 9). These results indicate that flavonoids and flavonoid biosynthesis might play a key role for tannin content in hyz sorghum.

## 4. Discussion

Sorghum, a crucial raw material for Chinese *baijiu* brewing, is rich in tannins. During microbial fermentation, these tannins decompose into typical aromatic substances that enhance the flavor of *Baijiu*. Among sorghum varieties, hyz, a specialized material of Chinese Maotai liquor, exhibits the highest tannin content within the five sorghum varieties used in this study. Comparative metabolomics was thus used to elucidate the metabolic mechanisms underlying the high tannin content in hyz.

In this study, KEGG enrichment pathway analysis revealed the metabolic pathways responsible for the quality differences between hyz and four other sorghum varieties. The flavonoid metabolic pathway exhibited the highest differential metabolites, suggesting its significant role in the high tannin content in hyz. Analysis of the KEGG metabolic pathways in four comparative groups indicated that the biosynthesis of secondary metabolites primarily accounts for the pigment differences among sorghum varieties. Key metabolic pathways include flavonoid biosynthesis, flavone and flavanol biosynthesis, and ABC transporter (Fig 5). Previous research has shown that ABC transporters are involved in the transport and accumulation of plant secondary metabolites (e.g., flavonoids, terpenoids, and alkaloids). Similar correlations have been also observed in other sorghum varieties between ABC transporters and grain color accumulation in metabolomics studies [31].

Existing studies have shown that flavonoids play an important role in sorghum resistance to stress. The integration analysis of transcriptome and metabolome revealed that sorghum roots respond to cadmium stress by regulating flavonoid biosynthesis pathways [8]. Metabolomics also revealed the differential metabolites expression of phenylpropanoid pathway regulating the defense of plant hormones of sorghum against aphid (*Spodoptera gracilis*) attacks [32,33]. Flavonoids, produced through the phenylpropanoid pathway, play a vital role in plant resistance to biotic and abiotic stress. Elevated flavonoid levels under salt-alkali stress can enhance salt tolerance in sorghum [34]. The total phenolic content varies in grains of different sorghum varieties, corresponding to differences in antioxidant capacities [35]. Analysis of metabolite compositions in various grains, including sorghum, reveals differences in nutritional value due to specific flavonoids and carotenoids [36]. Just to sorghum, the main differential metabolites among varieties include flavonoids, phenolic acids, and lipids, indicating genotype specificity. The hyz sorghum exhibits excellent brewing characteristics due to its higher amylopectin and tannin content, alongside with lower protein, lipid, and crude fiber content. This study indicates that the hyz contains high levels of phenolic compounds, although its high antioxidant capacity does not necessarily reflect its superior brewing qualities. Qualitative and quantitative analyses of individual compounds are needed to understand the metabolic basis of high tannin content in hyz. It had been reported that there is a trade-off between sorghum polyphenol content and volatile fatty acid derivatives, with *Tannin1* playing a central role [37]. Tannin-free sorghum shows reduced participation in flavonoid synthesis pathways and lower metabolite accumulation in anthocyanin and proanthocyanin biosynthesis. The loss of *Tannin1* function enhances volatile fatty acid derivative production, attracting bird predation. As high-tannin germplasm, metabolic data in hyz showed upregulation of DMAs in the flavonoid synthesis pathway, indicating it as the primary pathway controlling tannin content. Meanwhile, the lipid content in hyz is generally downregulated, reflecting a feedback regulatory mechanism of lipid metabolites on tannin synthesis. Tannin1 is a homolog of TTG1, composing a MBW complex with TT2, and TT8, which is essential for proanthocyanidin biosynthesis in *Arabidopsis thaliana* [38]. In the embryos of *ttg1* mutants, long-chain fatty acids (FAs) accumulated as a result of TTG1 indirectly downregulating genes such as GL2, a crucial negative regulator of FA biosynthesis in *A. thaliana.* Tannin1 negatively regulates the levels of FAs, subsequently leading to a decrease in downstream FA-derived volatiles in sorghum. This suggests that Tannin1 in tannin-rich sorghum may exert extensive metabolic regulatory effects on multiple anabolic and catabolic pathways associated with lipid metabolism.

Omics techniques can identify key functional compounds at different growth stages of sorghum, facilitating the targeted cultivation of new varieties to maximize yield under restrictive conditions. For instance, through integrated omics methods, carbon allocation patterns had been revealed during sugar accumulation of sorghum internode [39]. Changes in transcription and metabolism due to decreased cinnamyl alcohol dehydrogenase activity was significantly related to internode development of sorghum [40]. Metabolomics studies on flavonoid metabolites in different colored sweet sorghum grains identified six flavanones, including homoestriol, naringenin, prunin, naringenin, hesperetin, and pinocembrin, as major contributors to color differences [8]. This study found significant upregulation of compounds like naringenin in hyz, highlighting their central role in tannin accumulation. Integrated analysis of metabolic, transcriptomic, and photosynthetic physiological parameters under post-flowering drought revealed that galactose controls photosynthetic activity by regulating stomatal closure [41]. Sorghum under abiotic stress conditions such as drought, salinity, and extreme temperatures undergoes metabolic reprogramming involving compounds like proline, polyamines, and flavonoids [42,43], while differential distribution of salicylic acid and betaine mediates salt tolerance of sorghum [44,45]. Metabolomics also revealed the *de novo* biosynthesis and catabolic mechanisms of the defense compound during early sorghum grain and seedling development [46–48]. Thus, metabolomics is an effective tool for studying crop stress resistance, and future research can adopt temporal and spatial resolution metabolomics to explore key metabolites and metabolic pathways further.

In this study, we analyzed the differential metabolites of five sorghum varieties at the post-harvesting stage. The metabolomic analysis in this study had identified that the flavonoid metabolic pathway is the most crucial, with ellagic acid-4-O-glucoside being a key metabolite to tannin synthesis in sorghum. These findings would provide a clue helping to explore key genes for tannin biosynthesis, which can be utilized to design sorghum varieties not only retaining excellent traits but also with expected tannin content.

## Supporting information

Table S1 All compounds screened by UPLC/Mass in the five sorghum varieties. (XLSX)

Table S2 DAMs between hyz and jinl. (XLSX)

Table S3 DAMs between hyz and jinnl. (XLSX)

Table S4 DAMs between hyz and lnh. (XLSX)

Table S5 DAMs between hyz and lz19. (XLSX)

Table S6 Information of the 384 DAMs in Veen analysis within the five sorghum grains. (XLSX)

Table S7 A total of 38 DAMs were shared by all the five sorghum varieties. (XLSX)
